# Myocardial fibrosis is associated with subsequent death and hospitalization for heart failure in obese adults

**DOI:** 10.1186/1532-429X-17-S1-M8

**Published:** 2015-02-03

**Authors:** Yaron Fridman, Timothy C Wong, Kayla M Piehler, Karolina M Zareba, James Moon, Martin Ugander, Daniel Messroghli, John M Jakicic, Uma Valeti, Chung-Chou Chang, Sanjeev G Shroff, Christopher A Miller, Matthias Schmitt, Peter Kellman, Javed Butler, Mihai Gheorghiade, Erik B Schelbert

**Affiliations:** 1 Cardiology, Heart and Vascular Institute, UPMC, Pittsburgh, PA, USA; 2 UPMC Cardiovascular Magnetic Resonance Center, Pittsburgh, PA, USA; 3 University College of London, Heart Hospital Imaging Centre, London, UK; 4 Clinical Physiology, Karolinska Institutet and Karolinska University Hospital, Stockholm, Sweden; 5 Congenital Heart Disease and Pediatric Cardiology, Deutsches Herzzentrum Berlin, Berlin, Germany; 6 Cardiology Division, University of Minnesota, Minneapolis, MN, USA; 7 Biostatistics, University of Pittsburgh Graduate School of Public Health, Pittsburgh, PA, USA; 8 Bioengineering, University of Pittsburgh, Pittsburgh, PA, USA; 9 Centre for Imaging Sciences and Biomedical Imaging Institute, University of Manchester, Manchester, UK; 10 National Heart, Lung, and Blood Institute, Bethesda, MD, USA; 11 Cardiology Division, Emory University, Atlanta, GA, USA; 12 Center for Cardiovascular Innovation, Northwestern University Feinberg School of Medicine, Chicago, IL, USA; 13 Health and Physical Activity, University of Pittsburgh, Pittsburgh, PA, USA

## Background

Cardiac imaging in obese adults poses significant technical challenges, yet the prognostic value of diffuse myocardial fibrosis in obese adults quantified with cardiovascular magnetic resonance (CMR) extracellular volume fraction (ECV) measures is unknown. This issue is important because obesity increases the risks of death and hospitalization for heart failure (HHF). Myocardial fibrosis measured in obese adults with ECV may indicate vulnerability to death and HHF.

## Methods

We enrolled 480 consecutive obese patients with a BMI >30 referred for cardiovascular magnetic resonance (CMR) without amyloidosis, stress cardiomyopathy, or hypertrophic cardiomyopathy. We quantified myocardial fibrosis with CMR ECV measures in noninfarcted myocardium. Patient data, BMI, hematocrit, were collected on the day of CMR, and we tracked outcomes prospectively.

## Results

Median BMI was 35 (IQR 32-41), and median ECV was 27.7% (IQR 25.6%-30.9%, range). BMI and ECV were not related (p=0.90). Over a median 1.5 years (IQR 0.9-2.5yrs), 27 HHF events and 28 deaths occurred after CMR in 50 individuals. Adjusting for age, gender, renal function, myocardial infarction size, ejection fraction, hospitalization status, and heart failure stage, ECV in obese adults was associated with HHF (HR1.92 95%CI 1.40-2.65 for every 5% increase in ECV (ECV range: 16.6-45.8), death (HR 2.50 95%CI 1.59-3.95) or both (HR1.97 95%CI 1.44-2.70). ECV improved the classification of obese adults at risk and improved model discrimination for the composite outcome: e.g., HHF or death [continuous net reclassification improvement (NRI) 0.429, 95%CI 0.063-0.758; p=0.02; integrated discrimination improvement (IDI) 0.069, 95% CI 0.016-0.132; p=0.02].

## Conclusions

Despite the challenges of cardiac imaging in obese adults, diffuse myocardial fibrosis quantified by ECV is associated with HHF, death, or both. Myocardial fibrosis may represent a principal marker of cardiac vulnerability that improves risk stratification even in the setting of obesity. Since myocardial fibrosis can be reversible, myocardial fibrosis and the fibroblast that regulates it may be attractive therapeutic targets in obese patients.

## Funding

Dr. Schelbert was supported by a grant from The Pittsburgh Foundation,Grant M2009-0068, and an American Heart Association Scientist Development grant (09SDG2180083) including a T. Franklin Williams Scholarship Award; funding provided by: Atlantic Philanthropies, Inc., the John A. Hartford Foundation, the Association of Specialty Professors, and the AHA. Dr. Wong was supported by a grant K12 HS19461-01 from the AHRQ. Dr. Shroff's research was supported by the McGinnis Endowed Chair funds. This work was also supported by Grant Number UL1 RR024153 from the National Center for Research Resources (NCRR), NIH.

**Figure 1:**
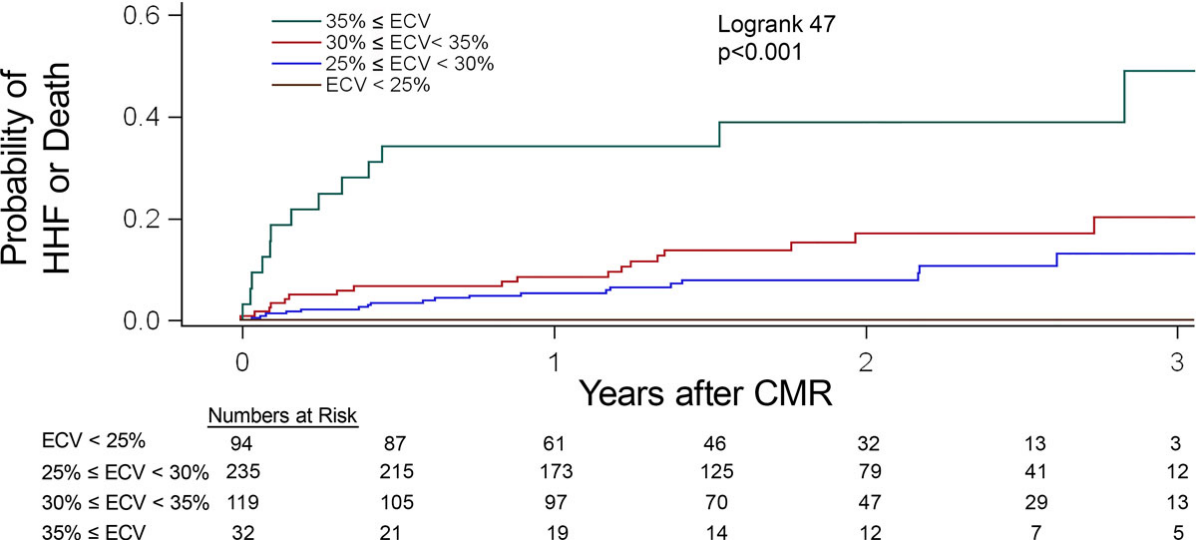


**Table 1:** In multivariable models, EC in noninfarcted myocardium in obese patients remained associated with the combined endpoint of HHF/death. The addition of ECV improved the classification of individuals at risk (new reclassification improvement, NRI) and the discrimination of the model (integrated discrimination improvement, IDI).

Multivariable Cox regression model	Hazard Ratio for every 5% increase in ECV (95% CI; p value)	Category free NRI (95% CI; p value)	Categorical NRI 0,.05, 0.10 risk categories (95%CI); p value)	IDI (95%CI; p value)
**Modeling composite of HHF or death,** stratified by heart failure stage and hospitalization status, adjusted for EF, age, glomerular filtration rate, myocardial infarction size, and gender	**1.97** (1.44-2.70; p<0.001)	**0.43** (0.06-0.76; p=0.02)	**0.04** (0.005-0.079; p=0.02)	**0.069** (0.016-0.132; p=0.02)

